# Proteome-Wide Differential Effects of Peritoneal Dialysis Fluid Properties in an In Vitro Human Endothelial Cell Model

**DOI:** 10.3390/ijms23148010

**Published:** 2022-07-20

**Authors:** Juan Manuel Sacnun, Robin Hoogenboom, Fabian Eibensteiner, Isabel J. Sobieszek, Markus Unterwurzacher, Anja Wagner, Rebecca Herzog, Klaus Kratochwill

**Affiliations:** 1 Division of Pediatric Nephrology and Gastroenterology, Department of Pediatrics and Adolescent Medicine, Comprehensive Center for Pediatrics, Medical University of Vienna, 1090 Vienna, Austria; n11927077@students.meduniwien.ac.at (J.M.S.); n12126271@students.meduniwien.ac.at (R.H.); fabian.eibensteiner@meduniwien.ac.at (F.E.); isabel.sobieszek@meduniwien.ac.at (I.J.S.); markus.unterwurzacher@meduniwien.ac.at (M.U.); anja.wagner@meduniwien.ac.at (A.W.); 2 Zytoprotec GmbH, 1090 Vienna, Austria; 3 Christian Doppler Laboratory for Molecular Stress Research in Peritoneal Dialysis, Medical University of Vienna, 1090 Vienna, Austria

**Keywords:** peritoneal dialysis, vascular damage, peritoneal dialysis fluids

## Abstract

To replace kidney function, peritoneal dialysis (PD) utilizes hyperosmotic PD fluids with specific physico-chemical properties. Their composition induces progressive damage of the peritoneum, leading to vasculopathies, decline of membrane function, and PD technique failure. Clinically used PD fluids differ in their composition but still remain bioincompatible. We mapped the molecular pathomechanisms in human endothelial cells induced by the different characteristics of widely used PD fluids by proteomics. Of 7894 identified proteins, 3871 were regulated at least by 1 and 49 by all tested PD fluids. The latter subset was enriched for cell junction-associated proteins. The different PD fluids individually perturbed proteins commonly related to cell stress, survival, and immune function pathways. Modeling two major bioincompatibility factors of PD fluids, acidosis, and glucose degradation products (GDPs) revealed distinct effects on endothelial cell function and regulation of cellular stress responses. Proteins and pathways most strongly affected were members of the oxidative stress response. Addition of the antioxidant and cytoprotective additive, alanyl-glutamine (AlaGln), to PD fluids led to upregulation of thioredoxin reductase-1, an antioxidant protein, potentially explaining the cytoprotective effect of AlaGln. In conclusion, we mapped out the molecular response of endothelial cells to PD fluids, and provided new evidence for their specific pathomechanisms, crucial for improvement of PD therapies.

## 1. Introduction

Renal replacement therapy with peritoneal dialysis (PD) utilizes hyperosmotic PD fluids, which are instilled into and drained from the peritoneal cavity in regular intervals. An osmotic or oncotic gradient facilitates removal of excess water, uremic toxins, and waste products to partially replace kidney function in patients with end-stage kidney disease [1]. Clinically used PD fluids provide adequate removal of small solutes and fluid by a combined continuous drainage and smaller-scale reabsorption via the endothelial–peritoneal barrier (consisting of the peritoneal membrane, the interstitial tissue, and the vasculature embedded within) and the lymphatic system [2]. PD fluids are bioincompatible due to their non-physiological glucose concentrations for fluid and toxin removal, and due to their buffer systems, typically consisting of sodium lactate and bicarbonate. In order to prevent the excess formation of toxic glucose degradation products (GDPs) during heat sterilization and storage, PD fluids need to either exhibit a mildly acidic pH (first generation single-chamber PD fluids) or markedly acidic pH that is neutralized before instillation into the patient (second generation dual-chamber PD fluids). By replacing glucose with the glucose-based polymer icodextrin as the osmotic agent, exposure to glucose and GDPs can be reduced. However, icodextrin-based PD fluids can only be used for one PD exchange per day due to adverse events, and despite advantages remain bioincompatible (low pH, lactate buffer, GDPs) [3,4]. These unique physico-chemical properties render PD fluids bioincompatible and are the main drivers of peritoneal inflammation, angiogenesis, and fibrosis [5,6]. Therapy with PD, especially long-term PD, leads to histopathological changes of the peritoneal membrane mainly affecting the mesothelial monolayer and the submesothelial tissue including arterial and venous vessels, and capillaries [1]. Mesothelial-to-mesenchymal transition (MMT) and submesothelial fibrosis and thickening are the best-described direct cellular effects of PD fluid exposure [7,8]. The progressive damage of the peritoneum often becomes clinically evident by faster peritoneal transport rates and declining ultrafiltration capacity, eventually resulting in technique failure [1,9,10].

PD introduces well-described vascular damage in the peritoneal membrane resembling diabetic vasculopathies [9,11,12]. The endothelial cells of these vessels function as the actual filter that regulates the peritoneal fluid transport during PD [6]. Comprehensive analysis of common and differential molecular alterations induced by the different available PD fluids and their specific properties has been extensively conducted in mesothelial cells but is missing for endothelial cells [5,9,10,11,12,13]. In endothelial cells, only a few selected individual parameters have been analyzed following PD fluid exposure, but a holistic untargeted analysis to decipher molecular changes has not been performed [5,14,15,16,17]. As it is not feasible to perform repeated peritoneal biopsies in chronic PD patients, experimental models must be utilized to study these chronic vessel changes and their molecular perturbations associated with PD within the peritoneal membrane. Recently, we were able to demonstrate the relevance of an in vitro model using primary human umbilical vein endothelial cells (HUVEC) for the in vivo situation by mass-spectrometry-based bottom-up proteomic analysis [18]. This model allows gaining valuable insights into biological processes and molecular perturbations in endothelial cells exposed to PD fluids on mechanistic levels otherwise not possible in vivo, especially in chronic PD patients.

Mass-spectrometry-based proteomics has been developed into a versatile and powerful analytical tool for life sciences supporting differential analytic aspects of detectable proteins. Proteomics not only provided novel and crucial insights into etiology, treatment, and prevention of kidney diseases but also in renal replacement therapies [19,20,21,22]. Here, we applied proteome analysis to systematically study molecular effects of PD fluids with different characteristics on endothelial cells.

Therefore, in this study we aimed to identify common and specific molecular mechanisms of different PD fluid properties, such as pH, GDPs, and colloid osmotic agents directly on the vasculature of the peritoneal membrane, within an established in vitro model, to improve the understanding of deleterious effects on the peritoneal membrane and to identify targets to improve PD patients’ outcomes.

## 2. Results

### 2.1. Alterations Induced by Different PD Fluids in Endothelial Cells

To investigate molecular responses and mechanisms of damage of endothelial cells by PD fluids, we chose an established in vitro model of primary endothelial cells exposed to different PD fluids. To systematically investigate the effects on a proteome-wide level, we applied a multiplex approach based on tandem mass tags (TMT), off-line fractionation of digested proteins, and bottom-up proteomics analysis, using high-resolution mass spectrometry. HUVEC from three different donors were exposed to the most commonly clinically applied PD fluids with different characteristics, as well as a lab-made, filter-sterilized, and therefore GDP-free fluid (Filter) (Table 1). Normal cell culture medium served as control condition. After exclusion of single-peptide identifications and removal of contaminants, we identified 7894 unique proteins in the raw data (Supplementary Table S1). Of those, 5855 had abundance values in all individual treatment conditions and were used for further analyses (Supplementary Tables S1 and S2). Comparisons between HUVEC exposed to PD fluids compared with control condition yielded different numbers of altered proteins when applying a standard significance threshold and correction for multiple testing (Benjamini–Hochberg (BH) adjusted *p* < 0.05) (Table 1 and Supplementary Table S2). The highest number of proteins with altered abundances (*p* < 0.01 BH corrected) was found in endothelial cells exposed to filtered fluid. While the numbers of upregulated proteins were comparable between GDP-containing single-chamber and filtered fluids (1026 vs. 955), almost 40% more proteins were found to be downregulated by filtered fluid (Table 1).

**Figure 1:**
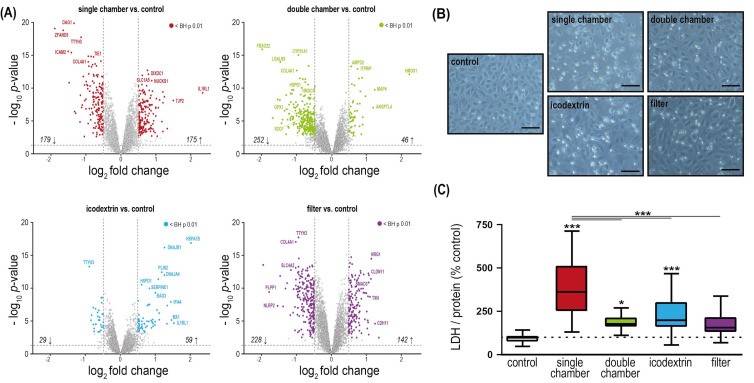
Change of endothelial cell proteome and morphology induced by PD fluid exposure. Primary human umbilical vein endothelial cells (HUVEC) from three different donors were exposed to standard clinically used PD fluids (single-chamber fluid: acidic pH, high concentrations of glucose degradation products (GDPs), an icodextrin-based PD fluid, and a filter-sterilized lab-made PD fluid based on the composition of the single-chamber fluid (filter). (**A**) Volcano plots for regulated proteins in HUVEC exposed to different PD fluids vs. control cell culture medium. *p* < 0.01 for all points above dashed line, colored points are *p* < 0.01(LIMMA) after Benjamini–Hochberg (BH) correction and log_2_[fold change] > 0.5 (**B**) Representative phase micrographs (magnification, ×20) of cells exposed to test fluids, scale bars = 100 µm. (**C**) Total protein-corrected lactate dehydrogenase (LDH) release into the cell supernatant (*n* = 3 individual experiments from three different donors, each comprising 6–9 replicate samples, means ± SEM). * *p* < 0.05; *** *p* < 0.001 (ANOVA, post hoc: Tukey’s).

The observation that the endothelial cell proteome is most strongly altered by filtered fluid is consistent when taking into account the magnitude of differential regulation (Figure 1A). HUVEC exposed to PD fluids showed morphological changes and cell detachment to a different extent (Figure 1B). Lactate dehydrogenase (LDH) release increased to 362% (±24%) (*p* < 0.0001) compared with control after single-chamber fluid exposure and following exposure to double-chamber fluid (177% ± 10%, *p* = 0.029), and icodextrin (198% ± 18%, *p* < 0.0001). LDH did not significantly differ from control when cells were exposed to filtered fluid (156% ± 17%, *p* = 0.09). Exposure to single-chamber fluids significantly elevated the LDH release not only in comparison with control, but also to all of the other PD fluids (*p* < 0.001) (Figure 1C).

### 2.2. PD Fluid Exposure Leads to Distinct Common and Fluid Specific Molecular Alterations in Endothelial Cells

**Figure 2:**
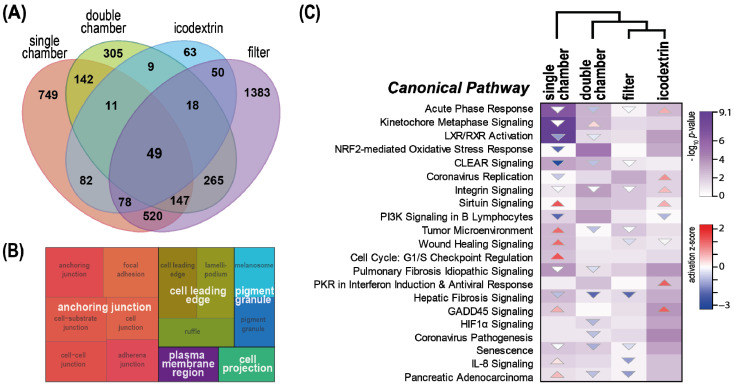
Common and differential effects of endothelial cells exposed to different PD fluids. (**A**) Venn diagram illustrating the overlap of significantly regulated (*p* < 0.01) proteins following PD fluid exposure. (**B**) Gene ontology (GO) cellular component analysis of the 49 proteins identified to be significantly regulated (Fisher’s exact test, *p* < 0.01 BH corrected) by all PD fluids. Only GO categories with log_2_ fold enrichment > 2 were further analyzed with the Revigo tool to summarize terms in a parental term, and the results are visualized as treemaps. (**C**) Heatmaps of significantly regulated canonical pathways (ingenuity pathway analysis, Fisher’s exact test *p* < 0.05) following PD fluid exposure compared with control medium. The *p*-value is visualized in violet by fill color intensity. Activation z-scores are indicated as up (activated; red) or down (deactivated; blue) colored arrows. The activation z-score is a calculated prediction of activation or inhibition of regulators based on relationships with dataset genes and direction of change of dataset genes. It represents the bias in gene regulation that predicts whether the upstream regulator exists in an activated or inactivated state. The direction of the arrow reflects the direction of the regulation (predicted activation: up, predicted inhibition: down).

We next characterized common molecular effects of PD fluid exposure in endothelial cells by focusing on the overlap exhibited by cells exposed to all four different kinds of PD fluids. On the individual protein level, 49 differentially regulated proteins (*p* < 0.01 BH corrected) were common to all four conditions (Figure 2A). Enrichment analysis of gene ontology (GO) cellular component terms was performed to describe the biological connotation of these proteins. The most enriched cellular component was anchoring junctions (Figure 2B). Three other terms involving the cell membrane (“cell leading edge”, “plasma membrane region”, and “cell projection”) were also found to be enriched. None of the different sets of proteins exclusively regulated by the individual PD fluids enriched these cellular component terms (Supplementary Figure S1), showing that this is a consequence of a core effect shared by all PD fluids. Pathway analysis of proteins differentially regulated by the individual PD fluids (i.e., all segments in Figure 2A), shows that 21 pathways were significantly enriched in all four exposure settings, but also showed considerable differences of their activation/deactivation scores (Figure 2C). Unsupervised hierarchical clustering of the pathway signature of the different PD fluids showed that filter and icodextrin-based fluid clusters (both have no or very low amounts of GDPs) and that the single-chamber fluid—the only condition combining acidic pH, high amount of GDPs, high glucose concentration, and lactate buffer—was most distant to the other fluids.

### 2.3. Specific Protein and Biological Processes Regulated by Individual PD Fluids

**Figure 3:**
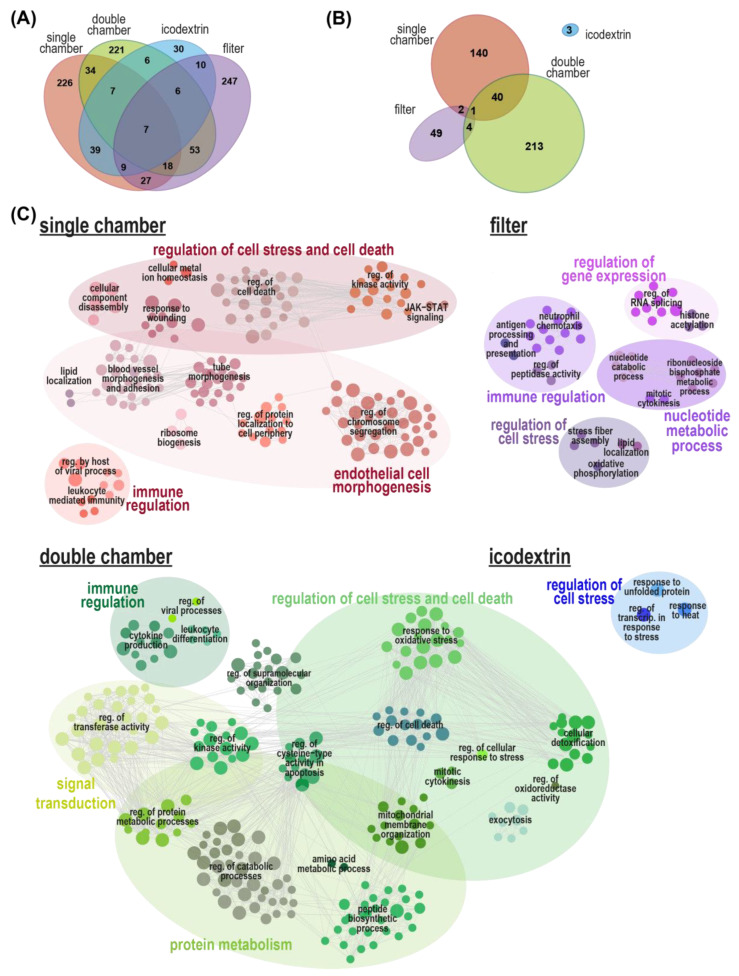
Enrichment maps of biological processes associated with proteins regulated exclusively by one kind of PD fluid. (**A**) Venn diagram illustrating the overlap of significantly regulated (LIMMA, BH *p* < 0.05, log_2_[fold change] > 0.5) proteins following PD fluid exposure. Only the proteins not overlapping were used for the biological process enrichment analysis. (**B**) Venn diagram illustrating the overlap of significantly enriched gene ontology biological processes analysis of each of the individual groups of proteins regulated by only one PD fluid. (**C**) Visualization of functionally grouped, significantly enriched (Fisher’s exact test *p* < 0.05) gene ontology (GO) terms of the non-overlapping significantly altered proteins by the individual PD fluids versus control. Network analysis of the enriched biological processes was performed with ClueGO in Cytoscape. Nodes were grouped according to their parent terms and connections. Node size represents term enrichment significance (smallest node: *p* < 0.05). Representative node labels are shown in each group, clustered in major biological processes. Non-connected terms were excluded from visualization.

Next, we analyzed the proteins that were regulated exclusively by individual PD fluids. To focus this analysis on specific perturbations triggered by a certain PD fluid, we introduced a fold change cut-off (log_2_[fold change] > 0.5) in combination with the significance threshold (*p* < 0.05 BH corrected), yielding a set of confidently altered proteins. Endothelial cells exposed to icodextrin-based PD fluid yielded the lowest number of differentially regulated proteins (30) compared with exposure to single-chamber (226), double-chamber (221), and filtered PD fluids (247) (Figure 3A). GO biological process enrichment analysis of these proteins only exclusively regulated by one of the PD fluid conditions yielded 260 significantly enriched terms (*p* < 0.05) for double-chamber PD fluids, followed by single-chamber (183), filtered (56), and icodextrin-based PD fluids (3) (Figure 3B). Biological process terms enriched by exposure to double- and single-chamber fluids shared 41 terms, while terms enriched by proteins only significantly regulated by icodextrin-based PD fluids were not shared with the processes enriched by the other PD fluids (Figure 3B). Comparison of the network analysis of the GO biological process terms enriched by each of the fluids compared with control medium showed that all four of them enriched parent terms related to cellular stress. Immune regulation was enriched in all conditions but icodextrin exposure. Exposure to single-chamber PD fluid was the only condition where endothelial cell morphogenesis was enriched. Filtered PD fluid specifically enriched the regulation of gene expression and nucleotide metabolic process. Exposure to double-chamber fluids led to protein regulation involving processes for signal transduction and protein metabolism (Figure 3C).

### 2.4. Major Properties of PD Fluids Perturb Common Proteins and Pathways but Not Always in the Same Direction

**Figure 4:**
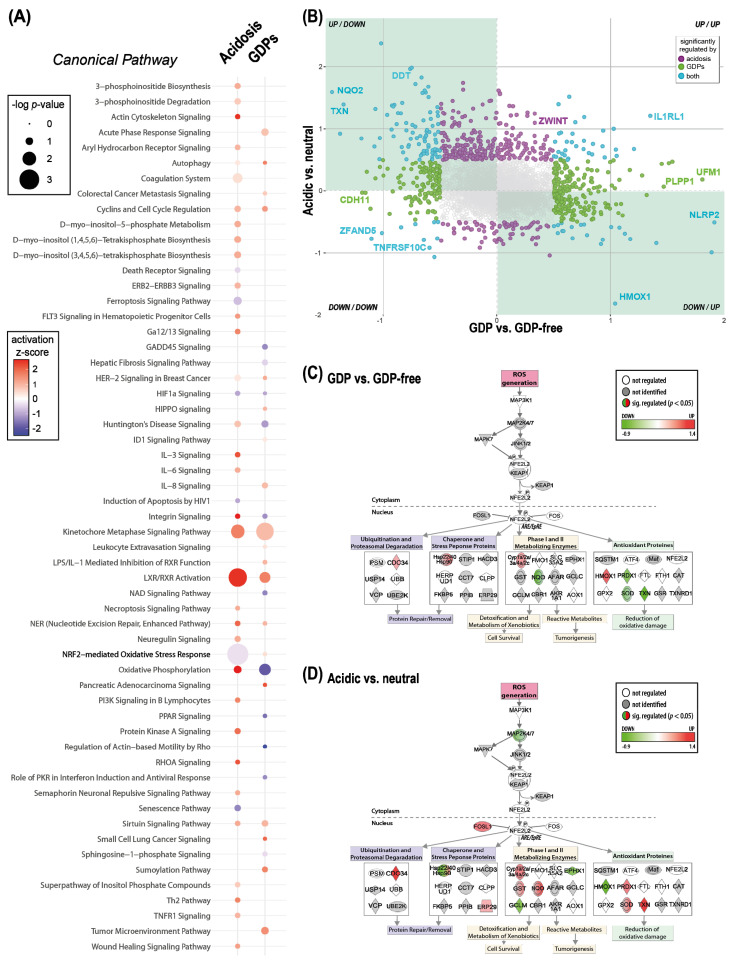
Effects of acidic pH or presence of glucose degradation products (GDPs) in PD fluids on endothelial cells. (**A**) Significant IPA canonical pathways (Fisher’s exact test *p* < 0.05) with an activation z-score ≠ 0; modeled acidosis effect: single-chamber, icodextrin, and filter versus double-chamber PD fluid; modeled GDPs effect: single-chamber, double-chamber, and icodextrin versus filter PD fluid; size of the nodes indicates the *p*-value of enrichment of the pathway. The activation z-score is a calculated prediction of activation or inhibition of regulators based on relationships with dataset genes and direction of change of dataset genes. It represents the bias in gene regulation that predicts whether the upstream regulator exists in an activated or inactivated state. Red: predicted activation, blue: predicted inhibition. (**B**) Co-regulation analysis of proteins significantly regulated (LIMMA *p* < 0.05) in either of the comparisons (green and purple) or in both comparisons (light blue). Comparisons: GDPs containing PD fluid vs. GDP-free and acidic PD fluid vs. neutral pH, log_2_[fold change] > 0.5 (**C**,**D**). The modified IPA NRF2-mediated Oxidative Stress Response Pathway overlaid with significantly (Fisher’s exact *p* < 0.05) regulated proteins in the comparison of GDPs-containing PD fluids vs. GDP-free (**C**) and acidic PD fluids vs. neutral pH (**D**). Down-regulated genes are shown in green and up-regulated genes in red.

Two of the main drivers of PD fluid-associated bioincompatibility are an acidic pH (of conventional single-chamber fluids) and varying content of GDPs. Following the analysis of the changes induced by individual fluids, we next analyzed the effects of the two major properties of bioincompatibility in a group-wise approach using a linear model. The different PD fluids were combined for the analysis according to their pH (acidic/neutral) and GDP content (GDP containing/GDP-free). We modeled the effect of acidosis by comparing the group of single-chamber, icodextrin, and filtered versus double-chamber PD fluids, and the effect of GDPs by comparing the group of single-chamber, double-chamber, and icodextrin versus filtered PD fluids. Pathway overrepresentation analysis (*p* < 0.05) of the GDP effect and acidosis effect yielded pathways that were enriched in both comparisons and showed the same direction of activation, such as autophagy, cyclins and cell cycle regulation, liver X receptor (LXR)/retinoic acid receptor (RXR) activation, and hypoxia-inducible factor 1-alpha (HIF1a) signaling. Pathways enriched in both comparisons but with opposing activation scores were integrin signaling, nuclear factor erythroid 2-related factor 2 (NRF2)-mediated oxidative stress, and oxidative phosphorylation (Figure 4A). The analysis also yielded pathways which were enriched only when GDPs were present, such as proinflammatory interleukin 8 (IL-8) signaling and acute phase-response signaling. While wound healing signaling, tumor necrosis factor receptor superfamily member 1a (TNFR1), and necroptosis signaling were only enriched under acidic conditions. Comparison of the modulation of individual proteins by GDPs or acidosis revealed that several proteins related to oxidative stress (thioredoxin (TXN), heme oxygenase 1 (HMOX1), nucleotide-binding site protein 1 (NACHT, LRR, and PYD domain-containing protein 2; NLRP2) were found as the most significantly regulated, though in opposite directions by the two stressors (Figure 4B). One of the master regulators of the response to oxidative stress in endothelial cells, NRF2, which was predicted to be inactivated with GDPs but activated with acidosis, is a representative example of how these two conditions have different targets and potentially mediate different responses in endothelial cells (Figure 4C).

### 2.5. PD Fluid Induced Perturbation of the Redox Stress Signaling Can Be Counteracted by the Addition of Alanyl-Glutamine

**Figure 5:**
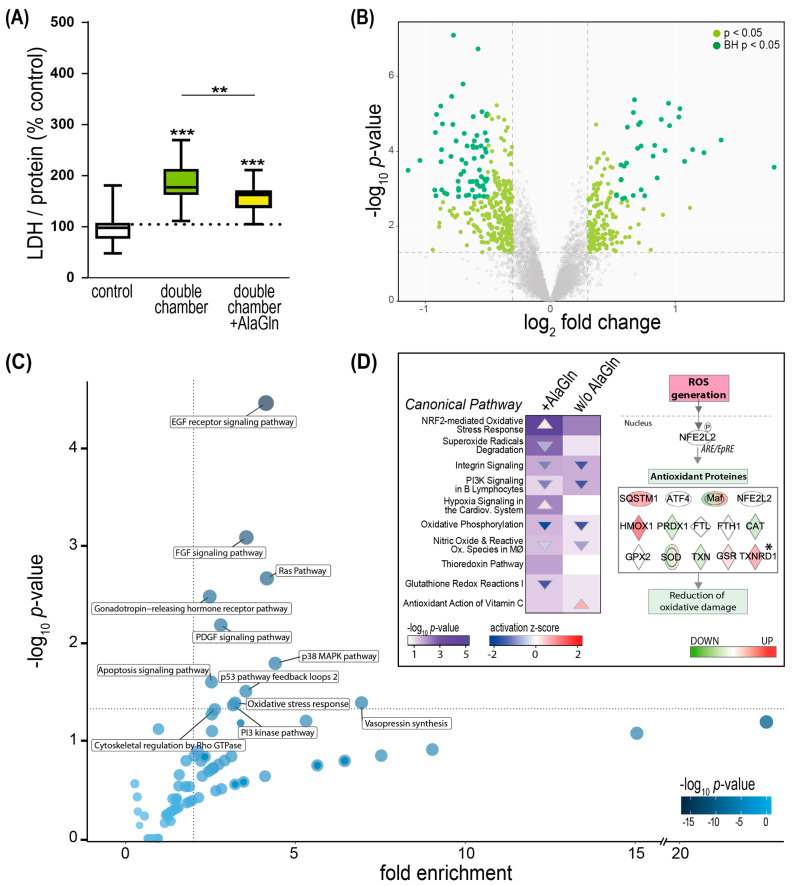
Effects of addition of alanyl-glutamine (AlaGln) to double-chamber PD fluids on endothelial cells. (**A**) Total protein-corrected lactate dehydrogenase (LDH) release into the cell supernatant (*n* = 3 individual experiments from three different donors, each comprising in total 18 samples, means ± SEM). ** *p* < 0.01; *** *p* < 0.001 (ANOVA, post hoc: Tukey’s). (**B**) Volcano plots for regulated proteins in HUVEC exposed to double-chamber PD fluid with added 8 mM AlaGln vs. double-chamber PD fluid without the additive. Colored points are LIMMA *p* < 0.05 and log_2_[fold change] > 0.3. Dark green: adjusted *p* < 0.05 after Benjamini-Hochberg (BH) correction for multiple testing. (**C**) Panther pathways analysis for the proteins regulated by AlaGln in double-chamber PD fluid. Each bubble represents a pathway, the intensity of the blue color is proportional to the *p*-value. Significantly enriched pathways are labeled with their respective names (Fisher’s exact *p* < 0.05, fold enrichment > 2). (**D**) Heatmap of significantly (Fisher’s exact *p* < 0.05) regulated IPA canonical pathways following double-chamber fluid exposure compared with double-chamber PD fluid supplemented with 8 mM AlaGln. Only oxidative stress related pathways are shown. The *p*-value is visualized in violet by fill color intensity. Activation z-scores are indicated as up (activated; red) or down (deactivated; blue) colored arrows. The direction of the arrow reflects the direction of the regulation (predicted activation: up, predicted inhibition: down). The NRF2-mediated Oxidative Stress Response pathway was extracted from IPA, manually curated and modified, and overlaid with differentially regulated proteins following exposure to double-chamber PD fluid with AlaGln vs. double-chamber PD fluid alone. Down-regulated proteins (green) and up-regulated proteins (red) * *p* = 0.042 (LIMMA).

We have previously shown that the addition of cytoprotective additives to PD fluid is a valid strategy to ameliorate the damage caused by PD fluids to peritoneal cells [11,18,23,24,25,26]. Alanyl-glutamine (AlaGln) has been described to counteract oxidative stress, so we next tested if it can ameliorate the observed effects of exposure of endothelial cells when added to double-chamber fluid (neutral pH). AlaGln addition to double-chamber PD fluid decreased LDH release, as a marker of cell damage, compared with standard double-chamber fluid exposure (155% ± 6 control vs. 185% ± 10 control, *p* = 0.0078) (Figure 5A). We further investigated the beneficial effect of AlaGln supplementation to double-chamber PD fluids and found 485 proteins regulated (*p* < 0.05 BH corrected) of which 187 were up- and 298 were down-regulated compared with exposure to the fluid without the additive (Figure 5B). Addition of AlaGln compared with exposure to double-chamber PD fluid led to enrichment of 12 pathways including oxidative stress response, mitogen-activated protein kinase p38 (p38 MAPK), and phosphoinositide 3-kinase (PI3K) signaling (Figure 5C). The addition of AlaGln to double-chamber PD fluid significantly enriched several oxidative stress-related pathways, including NRF2-mediated oxidative stress response, which was predicted to be activated compared with double-chamber PD fluid without AlaGln (Figure 5D). Thioredoxin reductase 1 (TXNRD1), an antioxidant protein and downstream effector in the NRF2-mediated oxidative stress response pathway, was significantly upregulated (*p* = 0.042) with the addition of AlaGln to double-chamber fluid compared with double-chamber PD fluid without AlaGln.

## 3. Discussion

Patients chronically treated with PD exhibit pathohistological changes within the vasculature of their peritoneal membrane. The observed vascular alterations and involved pathomechanisms have been described to resemble those seen in diabetes mellitus patients, and may ultimately lead to ultrafiltration failure [17,27,28,29,30]. The endothelial pathomechanisms induced by properties of clinically used PD fluids, which potentially lead to these clinically relevant alterations, have not yet been elucidated. We therefore first mapped molecular alterations of endothelial cells during PD fluid exposure using a proteomics approach and, second, identified perturbed cellular pathways and differential effects triggered by specific properties of PD fluids.

Currently used PD fluids induce progressive changes of the peritoneal membrane and its vasculature due to their distinct physico-chemical properties [31]. Despite the general agreement that all available PD fluids are bioincompatible for the cells present in the peritoneum, there is still a lack of understanding how PD fluids affect cell functions and perturb molecular mechanisms, leading to, if at all, only minor improvement in PD therapies over the last years [5,10]. Systematic comparison of PD fluids was performed mostly on clinical outcomes [32]. However, they failed to demonstrate clinically relevant differences in hard outcomes between different glucose-based PD fluids (single vs. double-chamber), likely due to few and underpowered clinical trials [32]. However, deeper phenotyping, such as performed in the pediatric biopsy registry, demonstrated marked peritoneal damage with severe vascular long-term changes [9,12,31,32,33]. We first analyzed effects commonly induced by all types of PD fluids and related them to known pathophysiological hallmarks of PD therapy. Proteomics analysis of the exposed endothelial cells showed that each of the PD fluids perturbed a high number of proteins. Icodextrin-based fluid exposure had the smallest effect on the endothelial cell proteome regarding the number of differentially regulated proteins. Even if numbers of perturbed proteins were comparable, such as single- and double-chamber bag fluids, the most significantly regulated proteins were not the same. Cell damage also varied with the different PD fluids, suggesting that each PD fluid might induce distinct responses. Filter-sterilized, GDP-free PD fluid resulted in the lowest amount of cell damage, comparable to control, despite affecting differential regulation of a number of proteins comparable to single-chamber PD fluid. This might be an example of how endothelial cells respond differently to properties of the two PD fluids. Despite the high numbers of proteins significantly regulated by the PD fluids, only 49 were affected by all tested PD fluids. Interestingly, the 49 proteins considered as the common PD fluid effect were enriched for cell junction-associated proteins. Endothelial cells represent the main barrier for the solute exchange during PD [4,19,34]. While transcellular transport of water through AQP1 accounts for up to 50% of the water transported during PD, ultrafiltration and barrier function are directly related to paracelullar transport, and cell junctions are the main regulators of this process [4,23,33,34,35,36]. We previously reported a significant cytoskeletal re-arrangement of HUVEC exposed to GDP-containing PD fluids, where major regulators of actin dynamics as well as of actin cytoskeletal structure were found to be disrupted [18]. Furthermore, cytoskeleton-associated proteins are involved in development of a senescent phenotype and alterations of junctions have both been described as effects of PD fluid exposure of mesothelial cells [21,26,37,38]. This common effect on one of the most important functions of peritoneal endothelial cells, the barrier function, could be directly linked to clinical PD technique failure, presented as loss of ultrafiltration or change of peritoneal transport rates and could be of high relevance regarding solutes transport during PD.

Other molecular pathways enriched by all PD fluids included acute phase response, integrin signaling, senescence, and fibrosis-related pathways. All of them—but especially fibrosis—are well-described pathomechanisms of the peritoneal membrane exposed to PD [7,37,38,39,40,41]. Different combinations of exogenous stressors (osmotic agent, pH, buffer system, toxic GDPs) which characterize the individual PD fluid properties also trigger more specific pathways. Consistent with the amount of cell damage, regulation of cell stress was found in all four PD fluids, while cell death regulation was found only in double- and single-chamber PD fluids. The fact that these processes are consistently enriched by all PD fluids, even though the proteins that lead to this enrichment are completely different, suggests that endothelial cells react to different PD fluid properties using different sets of proteins, yet resulting in similar higher level effects.

Some of the strongest-regulated proteins following PD fluid exposure were related to oxidative stress such as ribosyldihydronicotinamide dehydrogenase, thioredoxin, heme oxygenase 1 (HO-1), and NLRP2. This is also reflected on the pathway level, where we found two oxidative stress-related pathways to be enriched. However, pathways and proteins were regulated in opposite directions when we compared the differential effects of GDPs or acidosis. Our data therefore suggest that endothelial cells respond to oxidative stress induced by GDPs via HO-1.

Periodic instillation of acidic PD fluids, even though rapidly neutralized, still will repeatedly expose the peritoneal cells to acidic conditions. Cell death signaling, such as death receptor, ferroptosis, apoptosis, necroptosis, and senescence are overrepresented after exposure to acidic conditions. Several kinase signals were only triggered by acidosis such as ERB2, PI3K, and PKA signaling, potentially explaining the enrichment of the cell death-related pathways. Endothelial cells seem to respond to acidosis via superoxide dismutase (SOD), thioredoxin and peroxiredoxin-1. Oxidative stress is an important local and systemic pathomechanism in patients treated with PD. The high amounts of glucose itself have been shown to induce oxidative stress responses in mesothelial cells [40,42,43,44]. In addition, increased systemic oxidative stress arises already in early stages of chronic kidney disease, posing an important pathophysiological condition in PD patients [39,45,46,47].

We have previously shown that the addition of cytoprotective additives to PD fluid is a valid strategy to help to preserve peritoneal physiology on an experimental and clinical level [11,18,20,24,25,26,48,49,50]. Glutamine has been well described to have antioxidant capacity by supporting the glutathione-mediated redox potential [51,52]. AlaGln is a glutamine-releasing dipeptide, which is stable in solution. AlaGln has been shown in a randomized controlled clinical trial to have an anti-oxidative effect by reducing markers of oxidative stress in the peritoneal cavity when added to conventional single-chamber PD fluid [53]. In vitro AlaGln addition to single-chamber PD fluids regulated several of the pathways disturbed by PD fluid in endothelial cells [18,23]. The finding that cytoskeletal associated rho-GTPase represents one of the most strongly affected proteins and pathways extends our recent findings regarding effects of AlaGln on endothelial barrier function [18,23]. In this study, we investigated the effect of AlaGln addition to the more biocompatible double-chamber PD fluid, which is standard of care in most European countries. Among pathways regulated by double-chamber PD fluid, we identified the NRF2-mediated oxidative stress response. Among the proteins directly regulated by nuclear factor erythroid 2-related factor 2 (NFE2L2) are thioredoxin and thioredoxin reductase 1, which are part of one of the major antioxidant systems in mammalian cells [54]. Methylglyoxal is a well-described GDP in PD fluids and diabetes mellitus [12,55,56], and may impair the TXN/TXNRD1 and glutathione reductase (GSR) system in several cell types including aortic endothelial cells [57,58,59]. While the addition of AlaGln to double-chamber PD fluid did not affect the downregulation of thioredoxin compared to the exposure to double-chamber fluid without the additive, we found that AlaGln addition upregulated thioredoxin reductase 1 as well as glutathione reductase. This raises the hypothesis that AlaGln can counteract the GDP-induced decrease of thioredoxin by increasing thioredoxin reductase 1, thereby increasing the capacity of the remaining thioredoxin to detoxify the cells, leading to activation or deactivation of related pathways (i.e., apoptosis, p38) and improved survival. The discovery and the comprehension of these pathomechanistic changes introduced by PD into the otherwise mostly “dry” peritoneal cavity are of high importance to improve PD fluids.

The design of this study is inherently limited by the use of an in vitro HUVEC model, where exposure to PD fluids cannot actually reflect chronic PD. This in vitro system does therefore not consider the complex environment of vascular endothelium, especially as seen during uremia, where the vasculature is exposed to increased blood pressure and concentrations of uremic toxins [55]. However, the chosen primary HUVEC model recently showed itself to translate to vascular PD pathology, as HUVEC exposed to PD fluids demonstrated a large overlap of proteome and pathway expression with PD patient derived arteriolar tissue regarding shared pathways in structural proteins, cytoskeletal processes, and cell junctional proteins [18]. Importantly, this study aimed for differential decomposition of PD fluid properties, analyzing a complex biological system by a broad proteomics approach. With regards to the already complex analytical setup, a closely controlled, reproducible, and translational in vitro model was indicated. An additional level of variability introduced by a more complex experimental system would most likely decrease the analytical power. Finally, mechanistic insights might be limited by database bias, as current literature suggests that annotation pattern favors annotation growth towards already richly annotated genes, while dismissing potentially novel and under-researched targets in a self-perpetuating cycle [56]. We tried to minimize this bias by using different databases and bioinformatic pathway tools. Future studies investigating mechanistic effects of specific interventions targeting identified proteins and molecular processes perturbed in endothelial cells in more complex systems—i.e., barrier function studies—should be considered.

## 4. Conclusions

Well-defined perturbations of molecular processes and pathways during stress may represent highly attractive therapeutic targets to counteract endothelial cell injury. In this study, physico-chemical properties and their differential role on pathomechanistic effects of PD fluids were elucidated using an established in vitro model of human endothelial cells. Proteomics analysis of endothelial cells exposed to different PD fluids provided new insights of the differential effects triggered by the different composition of PD fluids, a key aspect to improve PD therapies. The cytoprotective additive AlaGln exhibited protective antioxidant action, associated with regulation of thioredoxin reductase 1, showing the potential of this approach to improve PD therapies. The obtained findings and raw data may serve as a resource for the PD community, focusing more and more on the role of PD-associated vasculopathy.

## 5. Materials and Methods

Standard chemicals were from Sigma-Aldrich (St. Louis, MO, USA) unless otherwise specified. Cell culture plastics were from TPP, Techno Plastics Products AG, Trasadingen, Switzerland.

### 5.1. Cell Culture

Primary human umbilical vein endothelial cells (HUVEC) from three different donors (Lonza, Basel, Switzerland; PromoCell, Heidelberg, Germany; ATCC-LGC Standards, Wesel, Germany) were cultured in 25 cm^2^ and 75 cm^2^ tissue culture flasks in endothelial basal medium (EBM-2, Lonza, Basel, Switzerland) containing 2% fetal calf serum (FCS) supplemented with endothelial cell growth supplements (EGM-2; Lonza) in humidified 5% CO_2_ at 37 °C. For experiments, cells from passages 2–5 were grown on 12-well plates.

### 5.2. Experimental PD Fluid Exposure Setting

HUVEC were exposed to experimental solutions for up to 24 h. All test fluids were sterile-filtered before usage. Each experiment consisted of three independent samples in biological replicates on separate culture plates and was repeated three times with cells from different donors. For PD fluid incubation, cells were first exposed for 40 min to pure PD fluid and subsequently exposed for 24 h to the PD fluid solutions diluted 1:1 with culture medium and brought to 2% FCS, or to normal medium without growth factors as control. The PD fluids used consisted of glucose-based high GDPs, acidic PD fluid (Dianeal PD4 3.86% glucose, Baxter, Castlebar, Ireland), low GDPs acidic icodextrin-based PD Fluid (7.5%, Extraneal, Baxter Healthcare GmbH, Vienna, Austria, manufactured in Castlebar, Ireland), low-GDP and neutral PD fluid (Physioneal-35 3.86% glucose, 1.75 mmol/L Ca, sodium bicarbonate and lactate buffer system, pH 7.4, Baxter Healthcare GmbH), or the same PD fluid supplemented with AlaGln dipeptide (8 mM, Dipeptiven; Fresenius Kabi, Bad Homburg, Germany), and lab-made, filtered, acidic GDP-free PD fluid: 3.86% glucose Sodium lactate-buffered, 1.75 mmol/L Ca, 0.22 µm filter-sterilized). All commercially available PD fluids were mixed immediately before use and sterile-filtered. Control cells were exposed in parallel to cell culture medium.

### 5.3. Cell Damage Assay

Cell damage following treatments of cells was assessed from lactate dehydrogenase (LDH) release into cell culture supernatants (TOX-7 LDH Kit, Sigma) per manufacturer instructions.

### 5.4. Protein Sample Preparation

Following experimental treatments, cells in 12-well plates were washed three times (250 mM sucrose, 10 mM Tris, pH 7.0) and lysed in 100 µL lysis buffer (30 mM Tris, 7 M urea, 2 M thiourea, 4% 3-[(3-cholamidopropyl) dimethylammonio]-1-propanesulfonate (CHAPS), 1 mM ethylenediaminetetraacetic acid (EDTA), one tablet of Complete Protease Inhibitor (Roche, Basel, Switzerland), and one tablet of Phosphatase Inhibitor (PhosSTOP, Roche) pH 8.5 per 100 mL). Total protein concentration was determined (Pierce 660 nm Protein Assay, Thermo Fisher Scientific, Waltham, MA USA) per the manufacturer’s manual. Lysates were stored at −80 °C until further processing.

### 5.5. SP3 and Tandem Mass Tag (TMT) Labeling

Between 50 and 70 µg of each sample and an internal pooled standard (IPS) consisting of equal parts of all samples were used. Digestion was performed using single-pot, solid-phase enhanced sample preparation (SP3). Briefly, the reduced (10 mM DTT for 1 h at 56 °C) and alkylated (55 mM IAA, 30 min at RT) proteins were bound to SP3 beads (10:1 beads:protein ratio, GE Healthcare), washed with 80% ethanol and acetonitrile, and subjected to on-bead digestion with trypsin/LysC (1:25 protease:protein ratio, Promega) overnight at 37 °C in 50 mM ammonium bicarbonate, pH 8.5. After elution peptides were desalted (Pierce Peptide Desalting Spin Columns, Thermo Fisher Scientific, Waltham, MA, USA), dried in a vacuum concentrator, and reconstituted in 100 mM TEAB, pH 8.5 (Fluka). Labeling was performed with tandem mass tag (TMT) 10plex or TMTpro 16plex (Thermo Fisher Scientific) according to the instructions provided by the manufacturer. TMT reagents were reconstituted with acetonitrile and each sample was labeled with 1 vial of TMT reagent. After incubation for 1 h at RT, the reaction was quenched by addition of 5% hydroxylamine in TEAB and further incubation for 15 min at RT. Eluates were dried in a vacuum concentrator after C18 clean-up (Pierce Peptide Desalting Spin Columns, Thermo Fisher Scientific), and reconstituted in 20 mM ammonia formate buffer, pH 10, before fractionation at basic pH. Two-dimensional liquid chromatography was performed by reverse-phase chromatography at high and low pH. In the first dimension, peptides were separated on a Gemini-NX C18 (150 × 2 mm, 3 µm, 110 A, Phenomenex, Torrance, CA, USA) in 20 mM ammonia formate buffer, pH 10 and eluted over a 32 min gradient from 0% to 30% solvent B, followed by 6 min at 100% solvent B at 50 µL/min using an Ultimate 3000 RSLC micro system (Thermo Fisher Scientific) equipped with a fraction collector. Fractions were collected every 30 s to a total of 36 fractions. Organic solvent was removed in a vacuum concentrator and samples were reconstituted in 5% formic acid or 0.1% trifluoroacetic acid. Fractions were analyzed at low pH on an Ultimate 3000 RSLC nano coupled directly to a QExactive mass spectrometer (both Thermo Fisher Scientific). Samples were injected into a reverse-phase C18 column (50 cm × 75 µm i.d., packed in-house) and eluted with a gradient of 6% to 65% mobile phase B over 94 min by applying a flow rate of 230 nL/min. MS scans were performed in the range from *m*/*z* 375 to 1650 at a resolution of 70,000 (at *m*/*z* = 200). MS/MS scans were performed choosing a top-10 method for peptide identification and relative quantitation of TMT reporter ions with the following parameters: resolution 35,000; normalized collision energy 33% (TMT10) or 30% (TMTpro); isolation width 1.2 *m*/*z*; dynamic exclusion 90 s.

### 5.6. Mass Spectrometry Data Analysis

The acquired raw MS data files were processed and analyzed using ProteomeDiscoverer (v2.4.0.305, Thermo Fisher Scientific). SequestHT was used as search engine and following parameters were chosen: database: Homo sapiens (SwissProt, https://www.uniprot.org/ (accessed on 24 September 2021)); enzyme: trypsin; max. missed cleavage sites: 2; static modifications: TMT6plex or TMTpro (K and peptide N-terminus) and carbamidomethyl (C); dynamic modifications: oxidation (M), acetyl (protein N-terminus), met-loss (M), and met-loss+acetyl (M); precursor mass tolerance: 10 ppm; fragment mass tolerance: 0.02 Da. For reporter ion quantitation, the most intense *m*/*z* in a 20 ppm window around the theoretical *m*/*z* was used. Correction of isotopic impurities for reporter ion intensities was applied. Only unique peptides were used for quantitation, which was based on *S*/*N* values with an average *S*/*N* threshold of 10. Normalization was based on total peptide amount and scaling mode on control averages with the internal standard as control. Only peptides and proteins with FDR < 0.01 are reported. Single peptide IDs were excluded from the dataset. Four proteins were classified as contaminants and excluded from further analyses.

### 5.7. Enrichment Map Analysis

Functional analysis of differentially abundant proteins was performed using Protein Analysis Through Evolutionary Relationships (PANTHER) version 17.0. The PANTHER analysis tool was used to perform enrichment analysis for the identification of over-represented biological pathways by a gene list. Annotation databases included in the analysis were Gene Ontology (GO) cellular component and PANTHER pathways. Cytoscape (v3.8.2) plug-in ClueGO (v2.5.8) was used to summarize and visualize functionally grouped terms of significantly enriched biological processes of significantly regulated unique genes with the following settings: database, Homo sapiens-GO_BiologicalProcess-EBI-UniProt-GOA; GO term fusion; on. Only nonredundant terms and significantly enriched pathways (*p* < 0.05) are shown, as well as the statistical test and two-sided hypergeometric test. GO terms are shown as nodes with links based on κ score (≥0.4), with at least four genes per term [60,61].

### 5.8. Statistical Analysis

Statistical analyses and graphical representations of results were performed using R (v4.0.3; http://www.r-project.org/ (released on 12 October 2020)) and Prism 9.4 (GraphPad, La Jolla, CA, USA). LDH release was normalized by sample protein concentration, expressed as mean ± standard error of the mean (SEM), and compared using ANOVA with post-hoc Tukey’s test. Comparisons between HUVEC exposed to PD fluids compared to control conditions yielded large numbers of differentially regulated proteins after multiplicity correction (Benjamini–Hochberg [BH]) at an alpha level of *p* = 0.05. While BH correction aids the minimization of Type I identification errors, and the wrongful exclusion of false negatives in the identified large and heterogenous amounts of proteins in this study was deemed unlikely, we applied a more conservative significance level threshold of 0.01. PANTHER GO enrichment analysis was performed utilizing the built in binomial test at an alpha of 0.05 BH. Pathway identification by Ingenuity Pathway Analysis (IPA 7.0, Qiagen, http://www.ingenuity.com (released in 30 April 2022)), their respective predicted up/down regulation patterns, and their affections by differentially abundant proteins were calculated for each functional pathway by a one-tailed Fisher’s exact test at an alpha level of 0.05. The IPA calculated z-score assessed the match of observed and predicted up/down regulation patterns and served as a predictor for the activation state. Differential protein abundances between PD fluid treatments were analyzed with linear models for microarray data (LIMMA) using the R package “limma” [62]. Limma is a combinatory statistical approach for large-scale expression studies fitting linear models for each gene/protein and utilizing Empirical Bayes and other shrinkage methods to borrow information across genes/proteins to stabilize the analysis and correct variance by shrinking it towards a pooled variance [62]. As mass spectrometry acquired proteomic data can be noisy, large, hierarchical in nature, and imbalanced due to acquisition and preprocessing methods, LIMMA, although being initially developed for microarray data, displayed superiority over conventional statistical modelling approaches (e.g., generalized linear models) [63]. Correction for multiple testing was performed by using the Benjamini–Hochberg procedure.

## Figures and Tables

**Table 1:** PD fluids properties and proteins significantly regulated following exposure versus control conditions.

PD Fluid	Osmolality (mOsm/L)	GDPs	pH	Osmotic Agent	Number of Proteins * (BH *p* < 0.05)			Number of Proteins * (BH *p* < 0.01)		
					All	Up	Down	All	Up	Down
**Single-chamber bag** (“Conventional”)	483	++++	~5.5	3.86% glucose	2468	1342	1126	1778	955	823
**Double-chamber bag** (“Biocompatible”)	483	++	~7.4	3.86% glucose	1677	787	890	946	406	540
**Icodextrin** (“Polyglucose”)	280–300	+	~5.5	7.5% icodextrin	726	274	452	360	126	234
**Filter** (“Lab made”)	483	-	~5.5	3.86% glucose	3232	1387	1845	2510	1026	1484

(*) differentially regulated following exposure, versus control condition. (++++) high; (++) moderate; (+) low; (-) none.

## Data Availability

Mass spectrometry data have been deposited into the ProteomeXchange Consortium (http://proteomecentral.proteomexchange.org) via the PRIDE partner repository with dataset identifiers PXD034985.

## Supplementary Materials

The following supporting information can be downloaded at: https://www.mdpi.com/article/10.3390/ijms23148010/s1.
