# Supplementary material for: Proteome-Wide Differential Effects of Peritoneal Dialysis Fluid Properties in an In Vitro Human Endothelial Cell Model
Source: Int J Mol Sci. 2022 Jul 20;23(14):8010. doi: 10.3390/ijms23148010 (PMC9317527; doi:10.3390/ijms23148010)
Supplement: Supplementary file 1 [file ijms-23-08010-s001.zip › ijms-1820826-supplementary Figure.pdf]

# Supplementary Material

## Supplemental Figure S1

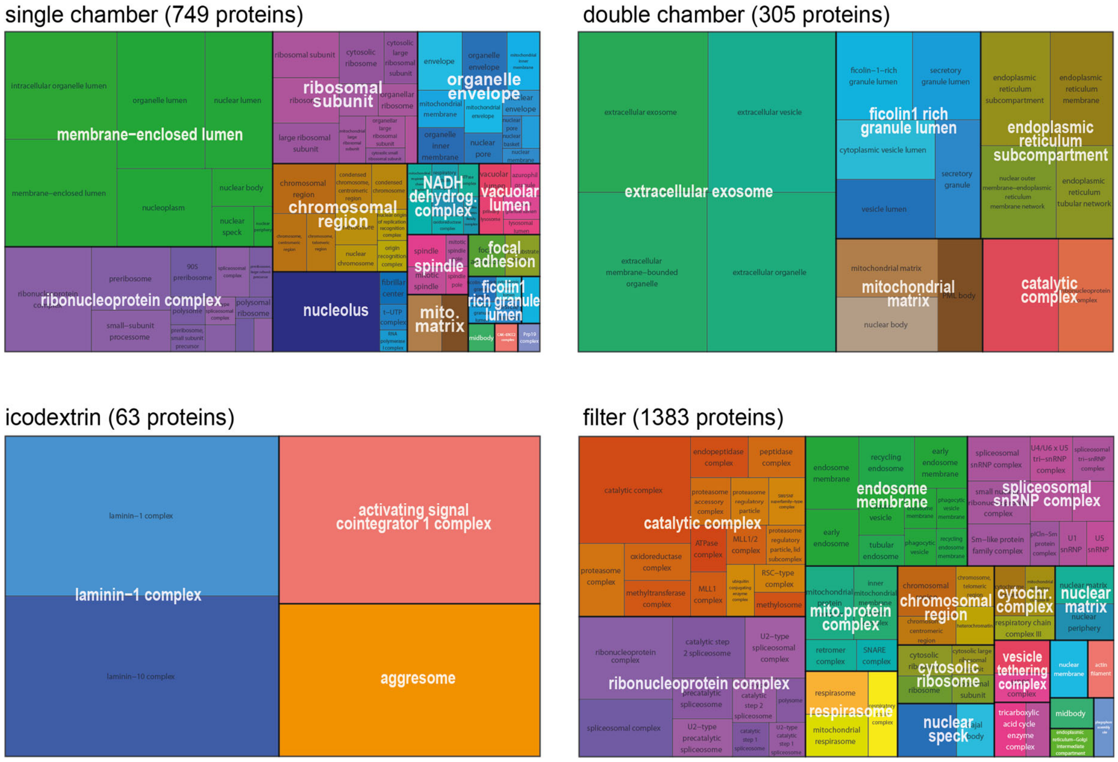


**Figure S1.** Gene ontology (GO) cellular component analysis of the proteins identified to be significantly regulated by individual PD fluids. Only GO categories with fold enrichment >2 were further analyzed with the Revigo tool to summarize terms in a parental term, the results are visualized as tree maps.
